# Investigation of network reorganization after epilepsy surgery is worth the effort

**DOI:** 10.1093/brain/awae172

**Published:** 2024-05-30

**Authors:** Lucas E Sainburg, Victoria L Morgan

**Affiliations:** Department of Biomedical Engineering, Vanderbilt University, Nashville, TN, USA; Vanderbilt University Institute of Imaging Science, Vanderbilt University Medical Center, Nashville, TN, USA; Department of Biomedical Engineering, Vanderbilt University, Nashville, TN, USA; Vanderbilt University Institute of Imaging Science, Vanderbilt University Medical Center, Nashville, TN, USA; Department of Radiology and Radiological Sciences, Vanderbilt University Medical Center, Nashville, TN, USA

## Abstract

This scientific commentary refers to ‘Connectome reorganization associated with temporal lobe pathology and its surgical resection’ by Larivière *et al*. (https://doi.org/10.1093/brain/awae141).

**This scientific commentary refers to ‘Connectome reorganization associated with temporal lobe pathology and its surgical resection’ by Larivière *et al*. (https://doi.org/10.1093/brain/awae141).**

Arguably the most effective treatment for drug-refractory focal epilepsy is surgical resection. The premise of resection of a seizure onset zone has its roots in the idea that focal epilepsy is, indeed, focal. However, non-invasive methods like functional and diffusion MRI have provided much evidence that focal epilepsy is a network disorder associated with widespread functional and anatomic alterations throughout the brain with implications for behaviour and cognition.^1^ Furthermore, a natural corollary is that a focal resection will also lead to changes across the brain beyond the resection site itself^2,3^ that may have significant behavioural and cognitive consequences.

But while investigations of presurgical networks in focal epilepsy are relatively common, far fewer studies attempt to examine the effects of surgery due to the added challenges of doing so. For a rigorous characterization of post-surgical alterations, both presurgical and post-surgical scans should be acquired for each patient (Fig. 1). This necessitates a more deliberate approach as the comprehensive imaging required for network quantification may not be routinely acquired after surgery on account of its having less direct clinical value. Heterogeneity in the patient cohort can add noise to presurgical investigations, but variability in surgical technique will add still further heterogeneity to a post-surgical sample. In addition, technical challenges such as presurgical to post-surgical image registration, segmentation of the resection cavity and anatomical shifting adjacent to the cavity, must be overcome. The benefit of this effort, though, may be a clearer understanding of the mechanisms supporting post-surgical behaviour and cognition that can be used to inform and improve surgical methodologies and outcomes. In this issue of *Brain*, Larivière and co-workers^4^ apply diffusion MRI and sophisticated dimensionality reduction methodologies to characterize global effects of anterior temporal lobectomy surgery and their relationship to clinical features and presurgical alterations in patients with temporal lobe epilepsy (TLE).

**Figure 1:**
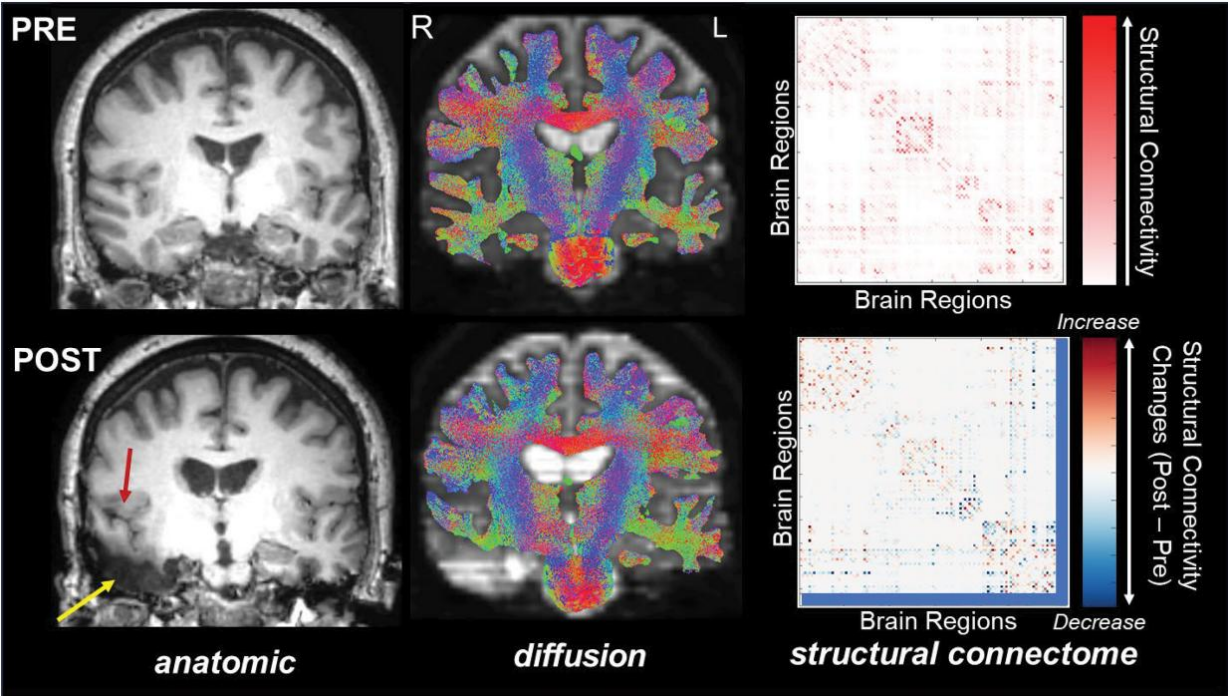
**Anatomic MRI (*left*), diffusion tractography (*middle*) and structural connectomes (*right*) acquired before and after right temporal lobectomy in a patient with unilateral temporal lobe epilepsy**. Anatomical images highlight the resection cavity (yellow arrow) and brain shift (red arrow) after surgery. The lower structural connectome highlights both connectivity increases and decreases from pre- to post-surgery. Blue bars indicate removal of resected regions from the connectome. L = left; R = right.

The study included 37 adults with drug-resistant unilateral TLE and 31 healthy control subjects. The surgical cavities were automatically segmented on the post-surgical images and manually edited to ensure that the extent of the resections was determined correctly. Whole brain network connectivity, referred to as the connectome, was quantified using diffusion MRI-based structural connectivity^5^ prior to, and a mean of 15 months after, surgery. To quantify the diffusion connectome changes, the authors implemented a methodology using cortical gradients^6^ that allowed the connectivity relationships to be represented in a space where regions with similar connectivity profiles are placed closer than regions with different profiles. In brief, this method resulted in four components or gradients representing a frontal versus temporo-occipital profile, an orbitofrontal versus sensorimotor profile, a medial occipital versus anterior temporal profile, and a sensory versus transmodal regions profile. After group changes relative to controls had been identified across each gradient, the results were further condensed to a four-dimensional Euclidean distance from the centroid of a set of gradients computed as a group template. Shifts away from the template were considered expansions of the connectome inferring segregation of connections, while shifts towards the centroid of the template were considered contractions or integration of regions.

To understand the interpretation of this sophisticated methodology, it helps to first consider the preoperative connectome compared to controls. This analysis showed significant changes in the ipsilateral hippocampus, contralateral amygdala and bilateral temporo-parietal and orbitofrontal cortices. The most significant changes were interpretated as increased segregation of the ipsilateral anterior temporal lobe and parahippocampal gyrus from the rest of the brain connectome, suggesting an isolation of the seizure onset zone. After surgery, there was evidence of reorganization leading to increased integration of the contralateral temporo-parietal regions with the rest of the brain, which may reflect compensation for loss of the homologous resected regions. Notably, presurgical to post-surgical changes were only detected in locations that showed significant preoperative changes relative to controls. Moreover, the degree of preoperative change versus controls correlated with the postoperative change from presurgery. Using a partial least-squares analysis, postoperative gradient changes were also found to be related to presurgical grey matter atrophy in subregions of the hippocampus, seizure frequency and secondary seizure generalization, but not to post-surgical seizure outcome.

This study is an important step in characterizing the effects of epilepsy surgery across the brain, but there remains much work to be done. Quantifying structural connectivity using the number of tractography streamlines between regions, as was done in the current study, has the added challenge of potential bias in the post-surgical data. This is because the same number of streamlines are seeded in the presurgical and post-surgical datasets, but there are fewer targets for the streamlines to reach in the post-surgical data. Thus, some regions may have an increased number of post-surgical streamlines that would have gone to other regions in the presurgical data, and it is not clear that this should be interpreted as an increase in connectivity. In this work connectomes were thresholded to retain only the top 25% of connections to minimize this bias, though there is presently no clear consensus on how to avoid this issue completely.

After computing MRI-derived connectomes, the analysis choices are vast and determine the types of questions that can be investigated. In this study, data reduction techniques were used to identify changes projected onto low-dimensional space (gradients) in a data-driven manner. This method may be specifically sensitive to global effects, but is inherently less directly interpretable on a regional, potentially more clinically understandable, level. For example, the authors propose that the segregation of the ipsilateral mesial temporal structures prior to surgery in the patients may relate to hyperconnectivity within the seizure onset zone and hypoconnectivity of this set of regions with the rest of the brain, as a compensatory mechanism to reduce the propagation of epileptogenic activity. However, these hypotheses could be more directly tested in future studies through analyses focused on specific connections. The results also showed overlap and correlation between the degree of presurgical (versus control) and presurgical to post-surgical connectome alterations, which the authors suggest may reveal a pre-existing vulnerability of these epilepsy-related regions to the effects of the surgical intervention. This idea can be more deeply explored by applying the surgical cavities to healthy control data to simulate the expected effects of a resection on a healthy brain, thereby eliminating those effects specifically related to the epileptic brain.^2^ This would provide insight into any potential biases of the gradient methodology in the context of resection.

Overall, the results presented by Larivière and colleagues^4^ provide clear evidence of global connectome shifts related to a focal structural disconnection. Their focus on white matter connections and their projection onto four major brain-wide gradients illustrates a general framework by which unexpected or indirect imaging-related consequences of a focal resection can be explained and compared to other physiological domains.^6^ Further work is needed to compare different resections, confirm how individualized patient phenotypes influence post-surgical changes, and relate post-surgical networks to outcomes. To do this will require individual sites and large-scale efforts, such as the ENIGMA consortium^7^ to collect and aggregate post-surgical data.
